# Saccade and Fixation Eye Movements During Walking in People With Mild Traumatic Brain Injury

**DOI:** 10.3389/fbioe.2021.701712

**Published:** 2021-11-05

**Authors:** Ellen Lirani-Silva, Samuel Stuart, Lucy Parrington, Kody Campbell, Laurie King

**Affiliations:** ^1^ Balance Disorders Laboratory, Department of Neurology, Oregon Health and Science University, Portland, OR, United States; ^2^ Department of Sport, Exercise and Rehabilitation, Northumbria University, Newcastle Upon Tyne, United Kingdom; ^3^ Northumbria Healthcare NHS Foundation Trust, North Shields, United Kingdom; ^4^ Veterans Affairs Portland Oregon Health Care System, Portland, OR, United States

**Keywords:** saccades, traumatic brain injury, gait, eye tracking, vision

## Abstract

**Background:** Clinical and laboratory assessment of people with mild traumatic brain injury (mTBI) indicate impairments in eye movements. These tests are typically done in a static, seated position. Recently, the use of mobile eye-tracking systems has been proposed to quantify subtle deficits in eye movements and visual sampling during different tasks. However, the impact of mTBI on eye movements during functional tasks such as walking remains unknown.

**Objective:** Evaluate differences in eye-tracking measures collected during gait between healthy controls (HC) and patients in the sub-acute stages of mTBI recovery and to determine if there are associations between eye-tracking measures and gait speed.

**Methods:** Thirty-seven HC participants and 67individuals with mTBI were instructed to walk back and forth over 10-m, at a comfortable self-selected speed. A single 1-min trial was performed. Eye-tracking measures were recorded using a mobile eye-tracking system (head-mounted infra-red Tobbii Pro Glasses 2, 100 Hz, Tobii Technology Inc. VA, United States). Eye-tracking measures included saccadic (frequency, mean and peak velocity, duration and distance) and fixation measurements (frequency and duration). Gait was assessed using six inertial sensors (both feet, sternum, right wrist, lumbar vertebrae and the forehead) and gait velocity was selected as the primary outcome. General linear model was used to compare the groups and association between gait and eye-tracking outcomes were explored using partial correlations.

**Results:** Individuals with mTBI showed significantly reduced saccade frequency (*p* = 0.016), duration (*p* = 0.028) and peak velocity (*p* = 0.032) compared to the HC group. No significant differences between groups were observed for the saccade distance, fixation measures and gait velocity (*p* > 0.05). A positive correlation was observed between saccade duration and gait velocity only for participants with mTBI (*p* = 0.025).

**Conclusion:** Findings suggest impaired saccadic eye movement, but not fixations, during walking in individuals with mTBI. These findings have implications in real-world function including return to sport for athletes and return to duty for military service members. Future research should investigate whether or not saccade outcomes are influenced by the time after the trauma and rehabilitation.

## Introduction

Evidence suggests that visual impairments may occur with mild traumatic brain injury (mTBI) (Ciuffreda et al., 2007; Capó-Aponte et al., 2012; Ventura et al., 2016). These impairments have the potential to affect functional capabilities in everyday life. For example, the visual system allows us to collect vital information about the environment required for safe navigation; it also plays a critical role in coordinating locomotion (Srivastava et al., 2018). In general, potential visual dysfunction of mTBI patients has been assessed using self-report and symptom-based outcomes (e.g. vestibular/ocular-motor screening, VOMS) (Mucha et al., 2014; Kontos et al., 2017; Whitney and Sparto, 2019). Although symptom-based outcomes of ocular motor performance are intended for aiding in concussion diagnosis, they may have limited ability to detect subtle deficits (Meier et al., 2015; Hunt et al., 2016; Snegireva et al., 2018). Comparatively, eye-tracking systems may detect and quantify subtle deficits in visual processes, especially with newer non-invasive technologies capable of sampling at the high frequency needed for capturing quick eye movements (i.e. 100 Hz) (Snegireva et al., 2018). While limited studies exist, this area of research has received increased attention over the last 2 decades (Pearson et al., 2007; Maruta et al., 2010b; Johnson et al., 2015; Snegireva et al., 2018), with impairment of eye-movement outcome measures in mTBI reported.

Most studies using eye-tracking systems with mTBI patients have been restricted to static/seated tests and with this testing paradigm, many differences in eye-movement between healthy controls and people with mTBI have been reported (Stuart et al., 2020a). For example, individuals with diagnosis of acute or post-acute (<12 weeks) mTBI have been found to have: 1) an increased pro-saccade error rate, and an increased saccadic reaction time latency during anti-saccade tasks (Balaban et al., 2016) in a battery of seated oculomotor, vestibular and reaction time tests.; 2) poor saccadic accuracy and longer response latency during horizontal and vertical saccade tasks on the performance of a vestibulo-ocular, visuo-ocular and reaction time battery of tests (I-Portal Neuro Otologic Test Center chair system) (Cochrane et al., 2019); 3) fewer saccades and more blinks compared with their baseline during a seated rapid number-naming task (King-Devick test) (Hecimovich et al., 2019); 4) greater gaze resultant distance, pro-saccadic errors and horizontal velocity in mTBI during a sport-like antisaccade postural control task (Wii Fit Soccer Heading Game) (Murray et al., 2017); 5) shorter time to first saccade, and greater intra-individual variability during a computer-based test performed seated (Suh et al., 2006); 6) longer anti-saccade reaction time at initial assessment (Webb et al., 2018), and greater anti-saccadic directional errors and lower gains in mTBI at initial assessment and follow-up in a visual stimulus test performed seated (Webb et al., 2018). Also, compared with healthy controls, mTBI patients have been found to have greater initial fixation error and greater accuracy error during the performance of a computer-based task (tracking of a circular target) (DiCesare et al., 2017). Although important, it is unclear how much these findings in static/seated tests relate to the demands involved in normal daily activity (Pelz and Canosa, 2001; Maruta et al., 2010a), including dynamic gait.

It is generally considered that gait impairments exist in mTBI populations, albeit some have found otherwise (Fino et al., 2018). For example, reduced gait speed was observed in patients in the acute stage, while stride length had mixed results across acute to subacute recovery stages. The most commonly reported deficit is gait speed, with mTBI groups walking slower in both single and dual task conditions (Fino et al., 2018). Gait requires integration of sensory systems information to be paired with the complex control and coordination of body segments for motor planning. Here, the visual system plays a critical role in the control of gait when all sensory information is available, and in cases of unreliable sensory information (Kennedy et al., 2003). Efficient locomotion is dependent on visual information gathered, specially, by saccades and fixation movements (Srivastava et al., 2018). It is plausible therefore, that deficits in eye movement may influence gait in a negative way. In such cases, we may expect to see an association between eye-tracking and gait metrics. To our knowledge this area has received little attention and is critical to understanding whether visual deficits impact real-world function in people with mTBI. Therefore, the aims of this study were twofold: 1) to evaluate differences in eye-tracking measures collected during gait between healthy controls and patients in the sub-acute stages (<12 weeks post injury) of mTBI recovery; and 2) to determine if there are associations between eye-tracking measures and gait speed in mTBI.

## Materials and Methods

### Participants

Data for this study were collected at two independent research sites: Oregon Health and Science University (OHSU) and Northumbria University (NU). Individuals with mTBI and healthy controls (HC) were recruited as part of an ongoing study at OHSU (ClinicalTrials.gov identifier: NCT03479541), while only HC participants were recruited at NU. For OHSU, approval of the study was granted through a joint Institutional Review Board from OHSU and Veterans Affairs Portland Health Care System (IRB # 17,370). At NU, the study was approved by the University Research Ethics Committee (REF: 23,365). Written informed consent was obtained prior to participation in the study from all participants. Sixty-seven individuals with mTBI and 37 age-matched HCs were enrolled in this study. For individuals with mTBI to be included, they had to: 1) be between 18 and 60 years old; 2) have a physician confirmed diagnosis of acute or post-acute mTBI (up to 12 weeks post-mTBI) based on VA/DoD clinical practice guidelines (Injury and Group, 2016). The medical diagnosis of all mTBI participants were double checked and confirmed based on their medical history or by our research team physician; 3) have no cognitive impairments that could interfere with task execution. People were excluded from the study if they: 1) had any other neurological or musculoskeletal condition that could explain motor dysfunction; 2) had moderate to severe substance-use disorder within the past month (Association, 2013); 3) had significant pain during the evaluation (7/10 by patient subjective report); 4) were currently pregnant; 5) had a past history of peripheral vestibular pathology or ocular motor deficits; 6) were unable to maintain 24 h without medications that may interfere with balance. Participants in the HC group must have had no cognitive impairments that could interfere with task execution, no diagnosis of concussion or any other condition that could influence variables assessed by this study. All participants had normal or corrected to normal vision (prescription lenses were worn during testing if required). Age, sex, height and mass were recorded for all participants. Days since injury and injury mechanism were also recorded for mTBI participants (Tables 1, 2).

**TABLE 1:** Demographic participant information.

Variables	Controls (n = 37)	mTBI (n = 67)	*p-* values
Age (Years)	32.40 (18.42)	32.85 (11.64)	0.012
Sex (M/F)	25/12	13/53	<0.001
Height (m)	1.76 (0.11)	1.69 (0.09)	0.001
Weight (kg)	85.98 (19.49)	73.55 (13.89)	<0.001
BMI	27.60 (4.84)	25.78 (5.15)	0.004
Days since mTBI (days)	—	43.23 (20.08)	—
Gait speed (m/s)	1.27 (0.16)	1.23 (0.17)	0.342

**TABLE 2:** Injury Mechanism of participants of mTBI group.

Injury mechanisms	Number of participants (n)	Distribution of cause of injury (%)
Sport-related	17	25.4
Motor Vehicle Accident	21	31.3
Fall	15	22.4
Bike	1	1.5
Other	13	19.4
Total	67	100

### Equipment and Experimental Procedures

Participants wore a mobile eye tracking system (head-mounted infra-red Tobii Pro Glasses 2, Tobii Technology Inc. VA, United States) and six inertial sensors (Opal v2, APDM Inc.) while performing a walking task. This protocol was based on previous studies investigating mTBI and other populations (Foulsham et al., 2011; Stuart et al., 2019b; Durant and Zanker, 2020; Stuart et al., 2020b). The Tobii system acquired the participant’s gaze coordinates through binocularly recording the participants pupils at a 100 Hz sampling frequency by means of infrared illumination. The inertial sensors consisted of tri-axial accelerometers, gyroscopes, and magnetometers, that measured segment accelerations, rotational rate and relative position at a 128 Hz sampling frequency. Inertial sensors were worn on both feet, sternum, right wrist, lumbar vertebrae and the forehead. Previous studies have shown that these sensors are valid and reliable for quantifying gait (Morris et al., 2019).

Participants walked back and forth over 10-m, at a comfortable self-selected speed. Each participant performed a single 1-min trial, which has been shown as an appropriate length of time to collect and conclusively interpret steady state gait measurements (Lord et al., 2013; Kribus-Shmiel et al., 2018; Kroneberg et al., 2018). Additionally, this length of test was shown to be logistically advantageous for pathological populations which may not be able to walk for extended periods of time (Nunes et al., 2017). The eye-tracker was calibrated prior to the walking task using the manufacturer’s single point calibration method. After the calibration and the walking trial started, participants were free to look wherever they wanted and begin walking. No instructions were giving to participants about where to look while walking as we aimed to look at real-world visual exploration (no structured visual requirements). Previous works have used the same task strategy (Stuart et al., 2015; Hunt et al., 2018; Drewes et al., 2021). When the walking trial finished, eye tracking data were stored within the Tobii system, and inertial sensor data were transmitted wirelessly to a nearby laptop for processing and storage. Participants at both sites (OHSU and NU) performed the same protocol, and both sites had similar laboratory spaces and setups when participants performed the 1-mintue walk.

### Data and Statistical Analysis

Raw eye tracking data (gaze coordinates–*x,y*) were extracted from the Tobii Pro Glasses 2 software and processed in Matlab using a custom-made validated velocity-based saccade detection algorithm (Stuart et al., 2014; Stuart et al., 2019b). Eye tracking outcomes included saccadic (frequency, peak velocity, duration and distance) and fixation measurements (frequency and duration). Gait velocity was calculated from the inertial sensors using the Mobility Lab software, V2 (APDM, Portland, OR, United States) (Morris et al., 2019). Gait velocity was selected as the primary gait outcome, because of its sensitivity in detecting gait differences between controls and individuals with mTBI (Fino, 2016; Howell et al., 2018).

Data were initially inspected for normality. Normality tests and inspection indicated a normal distribution for saccade frequency, saccade duration, saccade peak velocity and gait speed. Non-normal distribution was detected for age, height, weight, saccade distance, fixation frequency and fixation duration. Independent samples t-tests were used to compare differences between groups on demographic data with normal distribution, while Mann-Whitney U tests were used for non-normally distributed demographic variables. A Chi-Squared test was used to assess sex differences between the groups.

To test whether gait velocity and eye tracking outcomes differed between people with mTBI and HC, we fit a general linear model for each outcome. Demographic characteristics identified as significantly different between groups were used as co-variates in the models. Each model was adjusted for group (mTBI vs HC) and any co-variates. Initial models included group x co-variate interactions. If no group x co-variate effect was found at a 0.05 significance level, the interaction was removed from the final models.

The association between gait and eye-tracking outcomes within the mTBI group was explored using partial correlations controlling for demographic characteristics that were significantly different between groups. Only eye tracking measures that were significantly different between groups were used for correlation analysis. Statistical analyses were conducted using SAS 9.4 (SAS Institute Inc. Cary, NC, United States) and statistical significance was set at *p* < 0.05.

## Results

### Demographic Characteristics and Injury Mechanisms

Demographic characteristics are presented in Table 1. Injury mechanisms are presented in Table 2. The injury mechanisms included: sport-related (25.4%), motor vehicle accident (31.3%), injury caused by a fall (22.4%), bike accident (1.5%) and other general causes (19.4%).

There was no difference between groups for age (*U* = 1,005.500, z = −1.591, *p* = 0.112). There were significantly more males in the HC group than in the mTBI group (x^2^_(2,__*N=*104)_ = 23.994, *p* < 0.001). Participants from the HC group were significantly taller and heavier than the HC group (*U* = 621.00, *z* = −4.213, *p* = 0.001 and *U* = 722.00, *z* = −3.516, *p* < 0.001, respectively). As a result, we calculated body mass index (BMI) for participants and used it with sex as co-variates for the general linear models. Partial correlations were completed while controlling for sex and BMI. BMI was significantly lower for the mTBI group compared to the HC group (*U* = 810.00, z = −2.916, *p* = 0.004).

None of the models had significant group x sex, group x BMI, or group x sex x BMI effects. Therefore, results for the models for gait velocity and eye tracking outcomes focused on main effects for group, adjusting for sex, and BMI. There was no significant difference in gait velocity between mTBI and HC groups (*F*_(1,100)_ = 0.91, *p* = 0.342, partial *η* ^2^ = 0.009) after controlling for sex and BMI (Table 1 and Supplementary Table S1).

### Differences Between Groups for Eye Tracking Outcomes

**FIGURE 1:**
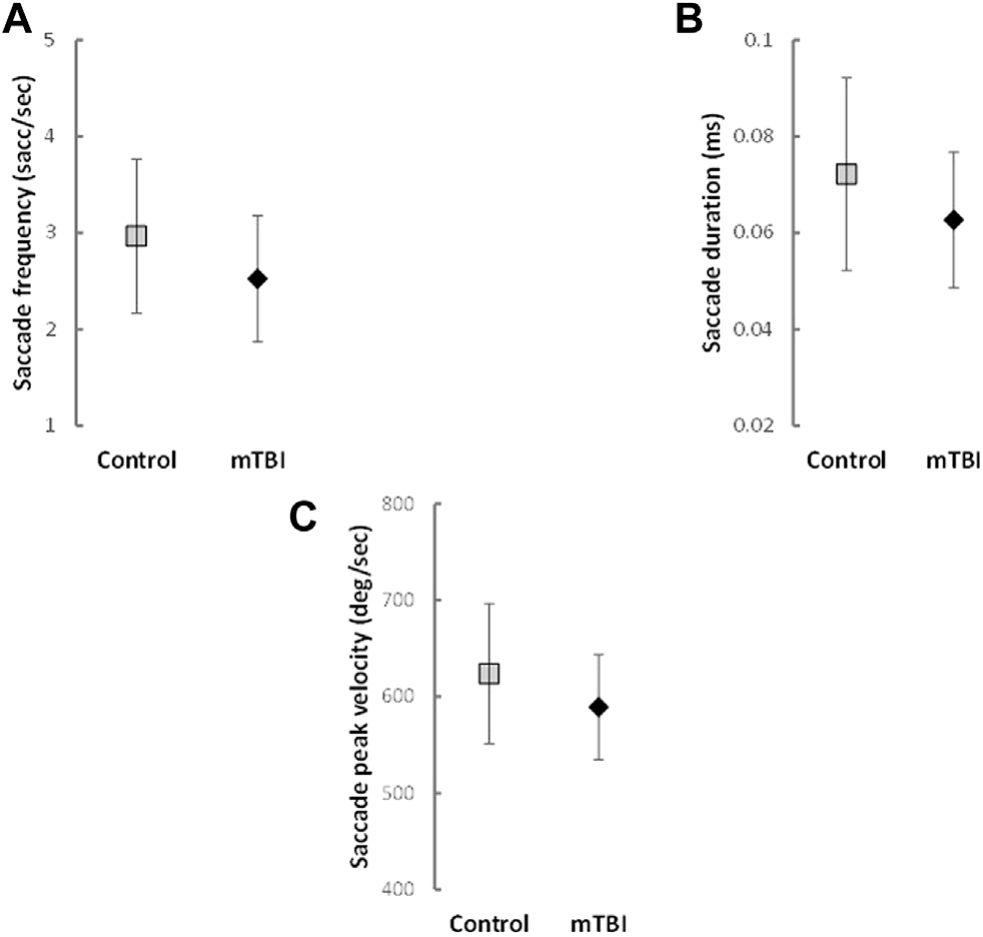
Means and Standard Deviations of saccade outcomes with significant differences between groups.

Analysis controlling for sex and BMI indicated that participants with mTBI had reduced saccade frequency (*F*_(1,100)_ = 6.05, *p* = 0.016, partial *η* ^2^ = 0.057; Figure 1A), saccade durations (*F*_(1,100)_ = 4.98, *p* = 0.028, partial *η* ^2^ = 0.048; Figure 1B) and saccade peak velocities (*F*_(1,100)_ = 4.71, *p* = 0.032, partial *η* ^2^ = 0.045; Figure 1C) compared to the HC group. No significant differences existed between groups for saccade distance (*F*_(1,100)_ = 0.12, *p* = 0.726, partial *η* ^2^ = 0.001), fixation frequency (*F*_(1,100)_ = 0.39, *p* = 0.531, partial *η* ^2^ = 0.004) or fixation duration (*F*_(1,100)_ = 1.64, *p* = 0.204, partial *η* ^2^ = 0.018). Parameter estimates for each general linear model can be found in Supplementary Table S1 and means and standard deviations in Supplementary Table S2.

### Correlations Between Eye Tracking Outcomes and Gait Velocity

**FIGURE 2:**
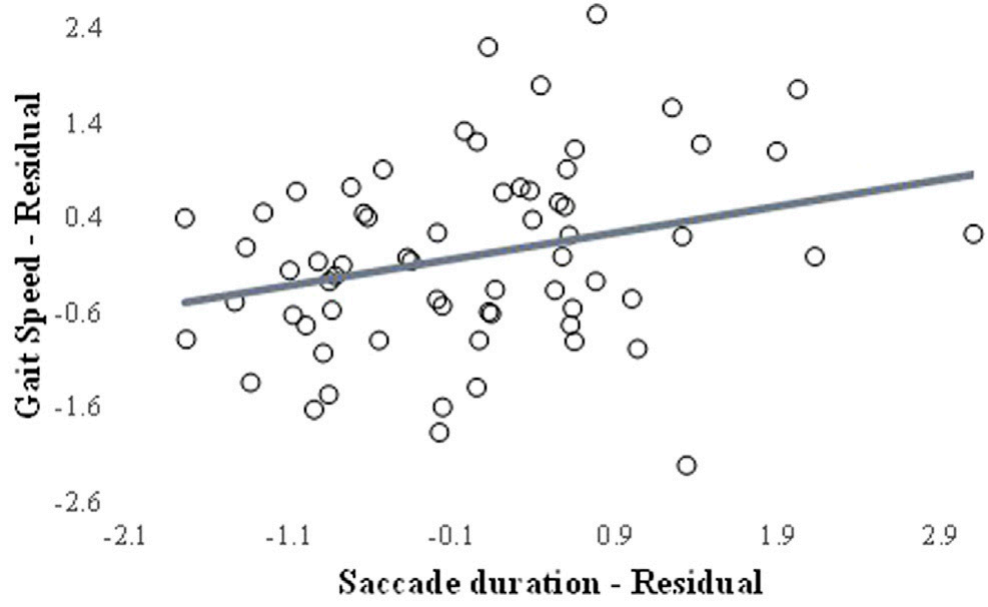
Scatter plot between the residuals of saccade duration and gait velocity.

A positive correlation was observed between saccade duration and gait velocity only for participants with mTBI. Specifically, participants with mTBI that had reduced saccade duration also had slower gait velocity (r = 0.287, *p* = 0.025; Figure 2). No other correlations between eye tracking outcomes and gait velocity were observed for either group (Supplementary Table S3).

## Discussion

This study compared saccadic and fixation eye movements during walking between people with mTBI and HC, and evaluated whether associations existed between eye-tracking measures and gait speed. Our main findings were: 1) participants with mTBI showed reduced saccade frequency, duration and peak velocity compared with an HC group; 2) reduced saccade duration positively correlated with slower gait velocity within the mTBI group. These findings suggest that mTBI affects eye movements while walking and highlight the importance of objective eye-tracking measures to better quantify mTBI impairments during tasks representative of daily life/function.

We observed deficits only for saccade outcomes, not fixations, in individuals with mTBI. Similar deficits in saccadic eye movements have been reported in older adults and other neurological populations while executing dynamic tasks such as walking (Dowiasch et al., 2015; Stuart et al., 2017). For example, reduced saccade frequency has been associated with deficits in cognition (Nelson et al., 2004) and slower saccade peak velocity has been found to be a predictor of attention decline in patients with Parkinson’s disease (Stuart et al., 2019a).The deficits we observed in saccadic function (i.e. reduced saccade frequency, duration and peak velocity) are outcomes that have been linked to attentional processes (Stuart et al., 2017). Attention has an important role on both walking (Morris et al., 2016) and saccadic control (Stuart et al., 2016; Ventura et al., 2016). Thus, we speculate that our findings indicate that mTBI participants may present reduced visuospatial attention while walking. Attentional deficits are commonly observed in people with persistent post-concussion symptoms and, although most of the cases resolve in 1 or 2 weeks, some cognitive impairments can last for up to 3 months–a timeline cohesive with our sample of mTBI participants (i.e. within 12 weeks of acute mTBI) (Rabinowitz et al., 2014; Wang and Li, 2016). Future studies should investigate the correlates between saccadic eye movements and cognition in tasks with greater visual demand such as obstacle avoidance, precision stepping, athletic and military tasks.

Saccade frequency is the basis of visual exploration (Kimmig et al., 2001), and the reduced saccade movements found herein may also indicate that patients with mTBI use a restricted exploration of the environment while walking. Dowiasch et al. (Dowiasch et al., 2015) suggested that changes in saccadic eye movements, such as diminished frequency and velocities, might be related to a narrow viewing strategy. This strategy could be explained by a greater effort being required for walking (Di Fabio et al., 2003) or less confidence in exploring the environment (Hadley et al., 1985), which could result from mTBI symptoms. The mTBI participants may have used a more restricted strategy of environment exploration to avoid exacerbation of mTBI-related symptoms, which can be provoked during ocular motor tasks. Considering that self-initiated eye movements can cause symptoms related to the mTBI (Mucha et al., 2014) it is possible that following an mTBI people voluntarily reduce saccadic eye movements in order to minimize symptoms, like dizziness and headache. However, to confirm that, future explorations of the relationship of saccade movements during walking and clinical symptoms of mTBI individuals are needed.

Although previous studies indicate an overlapping of saccade and gait control pathways (Srivastava et al., 2018), only saccade outcomes differentiated the two groups in this study, and we did not find differences between groups for gait velocity. These results are contradictory with some findings within the literature that indicate slower gait velocity in individuals with mTBI (Martini et al., 2011; Buckley et al., 2016; Fino, 2016; Fino et al., 2018). However, methodological aspects could explain the differences between studies including small sample size (Fino, 2016). Additionally, given saccades and locomotion are mediated by the integration of multiple brain areas and overlapping neural circuits (Srivastava et al., 2018), it is possible that participants with mTBI were allocating more attentional resources to walking instead of visual processing their environment. However, we were surprised that the only association between eye-tracking variables and gait velocity in the mTBI group were between saccade duration and gait velocity.

There is evidence of impaired sensorimotor integration in people with mTBI and balance deficits which may explain the impaired saccadic function. However, a recent study demonstrated that measures of sensorimotor integration, measured with the sensory organization (SOT) test, did not significantly relate to measures of saccadic accuracy, latency, and velocities measured during a seated target capture directed task (Campbell et al., 2021). Therefore, future studies would be needed to quantify relationships between sensorimotor integration for balance with context-free saccadic function during gait. It is important to highlight that our results are encouraging and suggest that eye tracking measures during walking, especially saccade outcomes, have potential for clinical application. These findings have implications for real world function, and measurements of saccades could be used as part of a diagnostic assessment for distinguishing mTBI from HC, as follow-up measures of intervention effects, as well as determination for return to sport for athletes and return to duty for military service members.

There is conflicting evidence on the utility of seated/static eye tracking measures in the assessment of deficits following mTBI (Balaban et al., 2016; Cochrane et al., 2019; Kelly et al., 2019; Stuart et al., 2020a). Seated or static eye-tracking evaluations allow for rigorous experimental control on evaluating the ocular motor system (Stuart et al., 2020a). However, the static evaluation position may allow for additional attentional resources to compensate for any deficits in ocular motor tasks following mTBI. In contrast, when people are navigating an environment while walking, deficits in ocular motor function may be more easily detected because of attentional demands required for not only visual processing but also motor coordination and cognitive processing tasks (Stuart et al., 2017; Stuart et al., 2020a). Therefore, eye tracking evaluation during gait may reveal more subtle impairments to gaze behavior not present during a static/seated evaluation. Further, there are some differences that may occur in dynamic laboratory testing compared to real world walking in the community (Foulsham et al., 2011). Future, studies should incorporate both static and dynamic evaluations, laboratory and real world, of ocular motor function to better understand the deficits caused by mTBI. For now, our work provides preliminary evidence of reduced saccadic function during walking after mTBI which is consistent with other work in a PD population (Stuart et al., 2017).

Although our results are promising, more studies are needed to identify different aspects that may contribute to changes in saccades during walking in mTBI individuals, including time since injury. For example, in a pilot study, Mullen et al. (Mullen et al., 2014) showed that individuals with mTBI presented changes in saccade movement, during a seated reaction time test 1 week after the injury, but not after 3 weeks. Our study included participants in post-acute mTBI stages (between 2 and 12 weeks) and so, results may have been impacted by the range of time after the initial concussion. It is plausible that those in the acute mTBI stage have more accentuated deficits in saccadic function while walking than those in post-acute stage. Future studies in this field should also investigate eye movements in a more complex environment including real world environments and dual task functions. The comparison between tasks that involve a higher motor control demand and static/sitting task should also be performed to verify if changes on eye movement control is task-related. Also, the inclusion of gait speed as the only gait characteristic measure may have limited some of our findings. Gait speed is a measure that essentially consists of an accumulation of more subtle gait characteristics (Morris et al., 2016) and may not detect subtle deficits in gait, and also may not correlate with eye movement deficits. Future studies should examine a comprehensive range of eye movement and gait characteristics (which will require greater numbers of participants) to explore subtle relationships that may exist as a result of underlying deficits. We limited gait metric comparisons between groups to gait velocity because of the variable’s ability to differentiate between groups from previous studies (Fino, 2016; Howell et al., 2018). However, other components of gait, specifically percent of time spent in double support, have also been shown to be different in those with chronic symptoms of mTBI (Cao et al., 2020). This variable is of particular interest because saccade frequency increases during the double support phase of gait. In our study, we are not able to identify the relationship between eye movement measures with specific gait phases as we did not have both eye-tracking and sensors, synchronized. Future studies should investigate not only a more comprehensive range of gait and eye movements characteristics, but also how specific phases of gait are related to eye movement control. Also, our findings do not allow us to indicate how specific neuronal circuits (or brain areas) are correlated with deficits in eye movement control in mTBI. Neural circuits that control eye movement include both cortical and subcortical regions of the brain and these circuits are widely distributed throughout the brain (Bigler, 2018). Many of these brain circuits are vulnerable to a concussive injury. Thus, future studies should propose protocols that include the data collection of eye tracking and neural activity/neuroimage, simultaneously, during real world tasks like walking. Data of this nature would allow a better understanding of how specific brain areas affected by the mTBI can be related with deficits in eye movement control.

## Conclusion

The current study demonstrated impaired gaze function in individuals with mTBI compared to HC by evaluating saccades and fixations during gait. Specifically, we found saccadic outcomes were impaired in mTBI during walking, with no changes between groups for fixation outcomes or gait velocity. Saccadic outcomes during walking have the potential to contribute to a better impairment characterization or diagnosis of individuals with mTBI, and perhaps aid in determining when a person can optimally return to complex activities such as sport or military service.

## Data Availability

The raw data supporting the conclusions of this article will be made available by the authors, without undue reservation.
