# Supplementary material for: Saccade and Fixation Eye Movements During Walking in People With Mild Traumatic Brain Injury
Source: Front Bioeng Biotechnol. 2021 Nov 5;9:701712. doi: 10.3389/fbioe.2021.701712 (PMC8602343; doi:10.3389/fbioe.2021.701712)
Supplement: Supplementary file 1 [file Table1.pdf]

Supplementary Material

Supplementary Figures

Supplementary Table S1. Parameter estimates for each general linear model of Control and mTBI.

Name	Unstandardized Slope Estimate	SE	*t*	*p*	Lower	Upper
Gait Velocity (m/s)						
Intercept	1.497	0.095	15.810	<.0001	1.309	1.684
Sex (Female)	-0.034	0.039	-0.870	0.388	-0.112	0.044
BMI	-0.008	0.003	-2.450	0.016	-0.014	-0.001
Group (mTBI)	-0.037	0.039	-0.960	0.342	-0.115	0.040
Saccade Frequency (number/s)						
Intercept	1.770	0.397	4.460	<.0001	0.983	2.557
Sex (Female)	0.062	0.159	0.390	0.698	-0.254	0.378
BMI	0.043	0.013	3.160	0.002	0.016	0.069
**Group (mTBI)**	**-0.394**	**0.160**	**-2.460**	**0.016***	**-0.712**	**-0.076**
Saccade Duration (ms)						
Intercept	0.040	0.009	4.490	<.0001	0.023	0.058
Sex (Female)	0.001	0.004	0.400	0.692	-0.006	0.009
BMI	0.001	0.000	3.700	0.000	0.001	0.002

**Group (mTBI)**	**-0.008**	**0.004**	**-2.230**	**0.028***	**-0.015**	**-0.001**
Saccade Peak Velocity (deg/s)						
Intercept	571.410	35.615	16.040	<.0001	500.751	642.070
Sex (Female)	-0.319	14.296	-0.020	0.982	-28.682	28.044
BMI	1.903	1.210	1.570	0.119	-0.497	4.303
**Group (mTBI)**	**-31.226**	**14.387**	**-2.170**	**0.032***	**-59.769**	**-2.683**
Saccade Distance (mm)						
Intercept	12.124	0.740	16.390	<.0001	10.656	13.592
Sex (Female)	-0.085	0.297	-0.290	0.775	-0.674	0.504
BMI	-0.033	0.025	-1.310	0.194	-0.083	0.017
Group (mTBI)	0.105	0.299	0.350	0.726	-0.488	0.698
Fixation Frequency (number/s)						
Intercept	1.057	0.345	3.060	0.003	0.371	1.744
Sex (Female)	0.001	0.128	0.010	0.993	-0.253	0.255
BMI	-0.018	0.012	-1.550	0.125	-0.042	0.005
Group (mTBI)	0.082	0.131	0.630	0.532	-0.178	0.343
Fixation Duration (ms)						
Intercept	205.813	21.199	9.710	<.0001	163.691	247.934
Sex (Female)	1.829	7.840	0.230	0.816	-13.750	17.407
BMI	-1.657	0.726	-2.280	0.025	-3.099	-0.214
Group (mTBI)	-10.323	8.065	-1.280	0.204	-26.349	5.702

* Significant difference between groups ($p < 0.05$)
