# Supplementary material for: Saccade and Fixation Eye Movements During Walking in People With Mild Traumatic Brain Injury
Source: Front Bioeng Biotechnol. 2021 Nov 5;9:701712. doi: 10.3389/fbioe.2021.701712 (PMC8602343; doi:10.3389/fbioe.2021.701712)
Supplement: Supplementary file 2 [file Table2.pdf]

Supplementary Material

Supplementary Figures

Supplementary Table S2. Means, Standard Deviations and *p*-values of the Control and mTBI groups.

Variables	Control	mTBI	*p*-values
Saccade frequency (sacc/sec)	2.96 (0.80)	2.53 (0.65)	**0.016***
Saccade duration (ms)	0.07 (0.02)	0.06 (0.02)	**0.028***
Saccade peak velocity (deg/sec)	623.83 (72.46)	588.99 (54.56)	***0.032****
Saccade distance	11.19 (1.35)	11.31 (1.23)	0.726
Fixation frequency (fix/sec)	0.55 (0.57)	0.67 (0.51)	0.532
Fixation duration (ms)	161.19 (43.59)	154.82 (27.19)	0.204

* Significant difference between groups ($p < 0.05$)
