# Supplementary material for: Saccade and Fixation Eye Movements During Walking in People With Mild Traumatic Brain Injury
Source: Front Bioeng Biotechnol. 2021 Nov 5;9:701712. doi: 10.3389/fbioe.2021.701712 (PMC8602343; doi:10.3389/fbioe.2021.701712)
Supplement: Supplementary file 3 [file Table3.pdf]

# *Supplementary Material*

## Supplementary Figures

**Supplementary Table S3.** Statistic values of partial correlation analysis.

| **Eye tracking variables** | **Gait speed** | |
|---|---|---|
| | Control | mTBI |
| Saccade frequency (sacc/sec) | r = 0.248, *p* = 0.222 | r = 0.149, *p* = 0.251 |
| Saccade duration (ms) | r = 0.072, *p* = 0.725 | **r = 0.287, *p* = 0.025*** |
| Saccade peak velocity (deg/sec) | r = 0.028, *p* = 0.891 | r = 0.104, *p* = 0.424 |

* Significant difference between groups ($p < 0.05$)
